# Intra- and Interspecific Differences in Diet Quality and Composition in a Large Herbivore Community

**DOI:** 10.1371/journal.pone.0084756

**Published:** 2014-02-24

**Authors:** Claire Redjadj, Gaëlle Darmon, Daniel Maillard, Thierry Chevrier, Denis Bastianelli, Hélène Verheyden, Anne Loison, Sonia Saïd

**Affiliations:** 1 Laboratoire d’Ecologie Alpine, Centre National de la Recherche Scientifique, Université de Savoie, Le Bourget-du-Lac, France; 2 Centre national d’Etudes et de la Recherches Appliquées sur la Faune de Montagne et les Cervidés-Sanglier, Office National de la Chasse et de la Faune Sauvage, Le Perray-en-Yvelines, France; 3 Chaire de recherche industrielle Produits forestiers Anticosti, Département de Biologie, Université Laval, Québec, Canada; 4 Systèmes d’élevage méditerranéens et tropicaux, Centre de coopération Internationale en Recherche Agronomique pour le Développement, Montpellier, France; 5 Comportement et Ecologie de la Faune Sauvage, Institut National de la Recherche Agronomique, Castanet-Tolosan, France; Ecole Normale Supérieure de Lyon, France

## Abstract

Species diversity in large herbivore communities is often explained by niche segregation allowed by differences in body mass and digestive morphophysiological features. Based on large number of gut samples in fall and winter, we analysed the temporal dynamics of diet composition, quality and interspecific overlap of 4 coexisting mountain herbivores. We tested whether the relative consumption of grass and browse differed among species of different rumen types (moose-type and intermediate-type), whether diet was of lower quality for the largest species, whether we could identify plant species which determined diet quality, and whether these plants, which could be “key-food-resources” were similar for all herbivores. Our analyses revealed that (1) body mass and rumen types were overall poor predictors of diet composition and quality, although the roe deer, a species with a moose-type rumen was confirmed as an “obligatory non grazer”, while red deer, the largest species, had the most lignified diet; (2) diet overlap among herbivores was well predicted by rumen type (high among species of intermediate types only), when measured over broad plant groups, (3) the relationship between diet composition and quality differed among herbivore species, and the actual plant species used during winter which determined the diet quality, was herbivore species-specific. Even if diets overlapped to a great extent, the species-specific relationships between diet composition and quality suggest that herbivores may select different plant species within similar plant group types, or different plant parts and that this, along with other behavioural mechanisms of ecological niche segregation, may contribute to the coexistence of large herbivores of relatively similar body mass, as observed in mountain ecosystems.

## Introduction

Body mass is one of the main determinants of the relative sensitivities of large herbivores to bottom-up (limitation by resource quality and quantity) and top-down (limitation by predation) processes [1]. Indeed, herbivores’ body mass correlates with metabolic requirements [2], [3], digestive capacity [4], and a suites of ecological traits such as openness of habitat used [3], [5], quality of the food resources consumed [6], [7], social structure [8],[9] and risk of predation [1], [10]. Interestingly however, the scaling of the different physiological (e.g. metabolic requirement, gut capacity) and ecological (e.g. intake rate) traits with body mass differs ([11], [3], reviewed in [12]), which has important ecological consequences, as it affects the capacity of species to exploit food of varying quality (larger species being able to eat more of low quality food and unable to select for small and sparsely distributed high quality food, while smaller species need to eat less in quantitative terms but need food of relatively better quality, which they are able to select for, [11], [3], [12]).

However, while body mass is positively related to the consumption of low quality food [2], [3], [4], [12], it does not, alone, predicts the range of food resources used by a herbivore [13]. A long-recognized ecological trait of herbivores is their relative consumption of browse *versus* grass [14], along in some cases, with the consumption of fruits [15]. Herbivore species are accordingly classified along a browser - grazer continuum [6] or in distinct categories (most often in 3 -browsers, grazers and intermediate feeders [16]-, and up to 7 such as in [15]). While there are few small-sized grazers and few very large sized browsers, body mass is, overall, a poor predictor of diet type [7]. The use of grass or browse, which greatly differ in nutrients, digestible fibers, and anti-herbivory compounds [17], [18], [19], can however be constrained by species-specific morphophysiological characteristics, related to forestomach anatomy, saliva quantity and composition and the level of stratification of rumen contents ([16], [20], [21], [22], [19]. Clauss et al. [20] have recently suggested to use a specific denomination for the gradient of rumen morphologies displayed by large herbivores, contrasting the “moose-type” to the “cattle-type” rumens at the two extremes. Stronger constraints seem to apply on “moose-type” species, which are suggested to be “obligatory non-grazers” [6] as they may avoid grass to a higher degree than “cattle-type” species avoid browse [6], [20], [23]. The first part of our study aimed at testing whether the diets of four large herbivore species (roe deer *Capreolus capreolus*, chamois *Rupicapra rupicapra*, mouflon *Ovis ammon* and red deer *Cervus elaphus*.) overlapping in geographic ranges differed in terms of composition and quality according to their differences in body mass (which should predict their consumption of low quality diet) and in types of rumen (which should predict the amount of browse/grass consumed and the diet niche width).

Connecting foraging processes to population dynamics, Illius and O’Connor [24] stressed the need to identify potential “food- key-resources” on which herbivores depend to survive for improving our understanding of herbivore regulation processes :“*given that the key factor determining animal population size is survival over the season of plant dormancy, key resources are those whose supply determines the size of the key factor*” ([24] p. 284). While the idea of key (food)-resource was developed in a single species framework, the identification of such key-resources is also relevant at a community level: when resources become limiting [25], competition can be expected strongest if species rely on the same plant species as key-resources. It raises the question of which resource, or which range of resources, is most important in the diet of large herbivores during the period of plant dormancy. However, connecting diet composition to demography in wild populations is challenging, which limits case studies where key-resources have been identified [26]. In addition, it is not self-evident that all herbivore species, even when sharing the same range, should rely on the same key-resource, given that species of different masses and/or with different rumen types are constrained in their selection of food items in different ways (see above). We can therefore hypothesise that identifying one or a restricted number of key-resources is more straightforward for small species/moose-type rumen species (that can not rely on low quality food/grass), than for large species/cattle-type rumen. Assuming that an indirect way to identify key-ressources is to find out which food items or a range of food items are best connected to diet quality in period of food shortage, the second part of our study aimed at quantifying the covariation between food composition and food quality at the intra- specific level during the period of plant dormancy, identifying potential key-ressources for each herbivore species, and testing whether the composition-quality covariation was related to herbivore’s consumption of low quality food (and hence to body mass) or to herbivore’s type of rumen (and hence to their ability to exploit alternative food items).

Our model community is a European large mountain herbivores community composed of four species which increase in numbers in the last decades has led populations to overlap increasingly in ranges, especially during autumn and winter [7]. With a few notable exceptions [27], [28], European large herbivore communities have been less studied than African communities [1], partly because they are less diverse, and partly because species overlap in space is recent [29]. Existing studies found a relatively large overlap in diet among species coexisting on the same mountain areas (*e.g.* in the Alps [27], [30], [31], [32]), but none has so far aimed at identifying species-specific key-resources by linking diet composition to diet quality at the intra-specific level. Collecting stomach samples during the hunting season is a unique opportunity to study species-specific diet characteristics during the period of food limitation. Our study here forth has therefore a double focus that should contribute to a better understanding of coexistence processes within communities of large herbivores: (1) testing whether rumen type and body mass account for differences in respectively diet composition and quality, and (2) testing whether the covariation between diet composition and diet quality allows identifying key resources, quality-wise, hypothesising that this covariation should be stronger for species of lower body mass and/or moose-type rumen. Additionally, our study provides new empirical data on wild ungulate diet and especially, on sources of variation of diet characteristics, which are valuable for meta-analyses (e.g. [15], [33]) and inter-specific comparisons aimed at linking morpho-physiology to trophic ecology (e.g. [34]).

## Materials and Methods

### Ethics Statement

All necessary permits were obtained for the described field studies. The Bauges Natural Regional Park (NRP) is managed by the Office National de la Chasse et de la Faune Sauvage (ONCFS), the Office National des Forêts and the NRP. The three institutions were part of and approved our research program. A specfic accreditation was delivered to the ONCFS to collect samples on animals legally shot by hunters (acreditation number 2009–2014).

Our study was based on samples collected by hunters on animals shot during the legal hunting season. All samples come from animals tagged with official annual hunting quotas delivered by the county prefect (prefectorial decree DDAF/SE#2004-231, #2005-250, #2006-140 235, #2007-177, #2008-135) in agreement with the environmental code (Art. R425-2 to 425-141 13). No animals were harvested for the sole purpose of this study.

### Study Area, Study Species and Data Collection

The study was carried out in the Bauges Natural Regional Park (NRP), a 81000 ha area located in the northern French Alps (45.65°N, 6.23°E), with an elevation ranging from 900 m to 2217 m. More than 70% of the NRP is covered by forests, mainly beech (*Fagus sylvatica*) and silver fir (*Abies alba*) on about 50%, the remaining areas being open pastures, screes and cliffs. The climate is cold and humid (annual mean temperature of 7.9°C, −1.1°C in January and 17.2°C in July on average, Météo France), with snow covering the ground from October to May, and frost during 148 days per year [35].

We studied three native ungulates (roe deer, red deer and chamois), and one introduced species (mouflon, released on the study site in the 1950’s, [36]), whose body mass, morphophysiology-based classification (“moose-type” to “cattle-type”) and diet-based classification (browser to grazer) are detailed in Table 1. Chamois and mouflon are most abundant in the South-East of the Park, while red deer are very abundant in the North-West part of the Park, wherefrom it colonised the remaining park range. Roe deer is distributed all over the study area (Figure S1 in File S1: maps with the distribution range of each species). The overlap in altitude ranges between animals harvested was large, though, as expected from the colonising history of each species, roe deer and red deer were on average at lower altitudes than mouflon and chamois (Figure S2 in File S1). During the study period (2003–2008), 576 roe deer, 464 chamois, 79 mouflon and 105 red deer on average were harvested annually.

**Table 1:** Classification of roe deer, chamois, mouflon and red deer according their body mass, digestive morphophysiology (“moose-type” to “cattle-type”) and diet category (browser to grazer).

	Body mass class	Digestive morphophysiology	Diet category
Roe deer	Small (∼25 kg)^a^	Moose-type^b^	Browser^c^
Chamois	Small (∼30 kg)^d^	Intermediate^e^	Intermediate^f^
Mouflon	Small (∼35 kg)^g^	Cattle-type/Intermediate^h^	Grazer/Intermediate^i^:
Red deer	Large (>100 kg)^j^	Intermediate^k^	Intermediate^l^

**References. ^a^:** [71], [72]; ^b^: [16], [73], [74]; ^c^: [54], [20]; ^d^: [75], unpublished data; ^e^: [16]; ^f^: [27], [76], [77]; ^g^: [75], unpublished data; ^h^: [74], [16], [73], [78]; ^I^: [33], [16], [76], [79], [74]; ^j^: [80]; ^k^: [81], [16], [73]; ^l^: [82], [76], [81].

From 2003 to 2008, 496 samples of rumen content were collected (from 1^st^ of September to the 31^st^ of January) and frozen until analysis on roe deer (n = 104), chamois (n = 148), mouflon (n = 86), and red deer (n = 158) and legally shot by hunters in Bauges NRP. Sex, age (Table S1 in File S1), body mass and geographic coordinates where recorded for most harvested animals.

### Determining Diet Composition

After mixing each rumen contents, 500 g sub-samples were washed in a 2 mm mesh sieve from which we selected 300 food fragments using a method adapted from the point-frame technique developed by [37] (see [38] for more details). Results were expressed as percentage of fragments for 104 items or plant groups identified to the lowest possible taxon using reference collections in each rumen. Each identified item was also assigned to a plant type among 18 categories: 1) forbs as any herbaceous dicotyledonous broad-leaved plants; 2) grasses as *Poaceae, Cyperaceae* and *Juncaceae* families; 3) legumes as *Fabaceae* family; 4) shrubs as woody plant with multiple stems and mature height <5–6 meters and 5) tree as woody plant with one primary stems and mature height greater than 5 meters. We also discriminated between 6) evergreen forb; 7) evergreen shrub; 8) evergreen tree; 9) fruit; 10) mushroom; 11) fern; 12) bryophyte; 13) lichen; 14) epiphyte; 15) bark; 16) dead leaf; 17) woody debris and 18) unknown items (see Table S2 in File S1).

The hunting season was split in two periods due to the presence of snow cover (usually present from the 15^th^ November): from 1st September to 14th November and from 15^th^ November to 31^st^ January, which are denoted as Period 1 (corresponding mainly to early fall) and Period 2 (corresponding roughly to early winter) in the following.

### Estimating Diet Quality

A total of 321 rumen content samples (28 roe deer; 100 chamois; 64 mouflon and 130 red deer), for which a sufficient quantity was available for analysis, were first dried at 60°C and then grounded with a cutting mill (Retsch SM100, Retsch GmbH, Hann, Germany) equipped with a 1 mm sieve. Samples were analysed using near-infrared spectroscopy (NIRS [39]). The samples were scanned on a monochromator reflectance spectrometer (FOSS NIRsystems 6500, Laurel, MD, USA) in small circular cups (50 mm) with a quartz glass cover. Spectral data were collected every 2 nm between 400 nm and 2500 nm. Each sample was scanned twice and spectra were averaged.

A subset of 118 samples chosen for the spectral representativity of the whole population was used to calibrate the spectra. For those, we measured Nitrogen content (N) using the Kjeldahl procedure and fibre fractions obtained by sequential fractionation [40]: NDF (neutral detergent fibre), ADF (Acid detergent fibre) and ADL (acid detergent lignin). These analyses allowed the estimation of the biochemical fractions of: hemicelluloses (NDF-ADF), cellulose (ADF-ADL) and lignin (ADL). The non-fibre fraction of samples (soluble fraction, SF) was calculated as (100-minerals-NDF; [16]). These reference analyses were used for the calibration of the NIRS by partial least square (PLS) regression [41] on the spectral data. Although animal species have different diets and therefore constitute subgroups in the rumen content database, a unique calibration could be performed because the groups appeared to widely overlap for chemical composition and spectral information. Rumen content database was gathered with wide (approx. 900 samples) CIRAD calibration databases containing plant materials comparable to the ones available to animals in our study. The resulting calibration equations had a R^2^ and standard error of 0.96 and 0.15% for N content, 0.94 and 3.40% for NDF, 0.95 and 2.50% for ADF and 0.95 and 2.06% for ADL. This level of precision is similar to the level obtained on plant material samples [42] and faeces [43]. Diet quality positively correlates with nitrogen and cell contents. Although digestible fibres (hemicellulose and cellulose) can contribute to the energetic content of the diet for species able to digest them, the overall diet quality should decrease with the total content of fibres, especially with lignin content, which is indigestible [18].

### Statistical Analyses of Diet Composition

We first described diet in terms of number of species eaten per period and diet niche breadth. Based on digestive morphophysiology, we expected species with intermediate types of rumen (red deer, chamois, and mouflon) to have a larger diet niche breadth than the species with a moose-type rumen (roe deer), which should avoid eating grass and low quality food items ([6], [34]), although empirical evidence on such patterns are equivocal [44], [27]. Following [44], we calculated the Shannon-Wienner information measure [45] of diet niche breadth per individual as B = −Σ(p_i_)ln(p_i_) where p_i_ is the proportion of item i in the diet. We tested for the effect of date and species on diet niche breadth using linear models, given that B was normally distributed (Pearson goodness of fit test: GOF = 30.04, p = 0.09).

Following [46] [47] and [44], we estimated species diet overlap using Schoener’s index of overlap [47] per pair of herbivore species and for each period, as O_jk_ = 1-0.5*Σ|p_ij_-p_ik_| where p_ij_ is the proportion of item i in species j and p_ik_ is the proportion of item i in species k. We expect overlap to be high among the three species with intermediate rumen type, and weak between the roe deer and the three other species [44], [27]. We performed a non-parametric Wilcoxon rank test to check whether the overlap changed from period 1 to period 2. As a consequence of the decrease in plant availability, we expected all herbivore species to consume similar plants to a higher degree in winter than in fall [44], and therefore to increase in overlap [44], [48].

We then performed a between principal component analysis (between-PCA, [49]) of the percentage items per plant type and rumen (see Table S2 in File S1) using herbivore species and period as factors. This allowed identifying the combination of plant types that maximise the differences between herbivore species and periods. Next, we tested whether the diet content in the 5 plant types that contributed most to the inertia, varied with date and herbivore species as well as their interaction, while accounting for year as an additive effect, using Generalized Linear Models ([50]). Date effect was measured by calculating the number of days since the 1^st^ of September. Hence, it was a continuous variable, which we modelled either with a linear or quadratic function (the latter to account for possible non-linear relationships of the consumption of a plant item with time). Variables under study were counts (number of food items belonging to a given plant type among the 300 food items sampled per rumen), which we modelled with negative binomial models, to account for overdispersion [51]. We selected the model with the lowest Akaike Information Criterion (AIC, [52]).

To test whether body mass or morphophysiological types accounted for inter-specific differences in diet content, we created 3 categorical variables. The first one, based on body mass (2 categories), opposed red deer as the heaviest species to the three smaller species. The second contrast was based on the rumen type classifications and opposed roe deer as a “moose-type” species, expected to be a browser, to mouflon, chamois and red deer as species with intermediate type of rumen. Last, we constrasted both roe deer and mouflon to the two other species. Compared to the former grouping, this tested the equivocal position of mouflon in the literature, where it has gone from being classified as a grazer to being now classified as an intermediate feeder (see [33] for a review). We substituted the species effect in the best model by one of this grouping and checked for a possible decrease in AIC.

### Statistical Analyses of Diet Quality and of its Relationship with Diet Composition

We analysed diet quality following the same steps as described above. We first performed a between-PCA [49] on rumen chemical content (nitrogen, lignin, hemicellulose, cellulose and soluble fraction) with herbivore species and period as combined factors (which effects were tested with a Monte Carlo test with 999 simulated partitions) to determine the covariation among chemical components that best contrasts species and period. Then, we defined a set of models per chemical component, testing for the effects of date and species and their interaction, while accounting for year as an additive effect, using linear models. As above, we substituted species effect by either body mass (2 categories) and the 2 variables according to morphophysiological type (3 categories or 2 categories), to test whether species-specific differences could be accounted for by one of these groupings. Models were selected based on AIC.

To estimate the covariation between diet quality and diet composition, we performed co-inertia analyses [53] between the PCA of diet composition (abundance per plant type in rumen) and the PCA of diet quality (chemical content), for each period and each species. The aim of the co-inertia analysis was to maximize the covariance between the diet composition and diet quality tables. The significance of the co-inertia coefficient (denoted RV) was tested using a Monte Carlo test with 999 simulated partitions. We expected that the more diverse a diet in terms of composition, the weaker the covariation between diet composition and diet quality. Accordingly, we expected roe deer to display the highest covariation between composition and quality, because it combines being small and having a moose-type rumen, which should constrain it to be the most selective species of all, especially during winter [54]. In contrast, the three species with an intermediate type of rumen (chamois, red deer, and mouflon) should be able to use a more diverse diet to maintain the highest possible diet quality, hence a weaker covariation between diet composition and diet quality. Among these latter species however, red deer, due to its large size, may have an even more diverse diet than chamois and mouflon, and therefore, exhibit the lowest covariation of all.

Given that the consumption of grass should be different among species, we tested whether the relationship of grass content to nitrogen, lignin, soluble fraction, cellulose and hemicellulose contents was species-specific [55]. Testing for a species-specific differences in the grass content to quality relationship implies testing whether the two way interaction between grass content and species effect is significant (the main effect of species only indicates a species-specific intercept). We logit-transformed grass content to get a non-bounded range of grass-content values, selecting only rumen with non-zero grass content. We then performed a linear regression with nitrogen, lignin, soluble fraction, cellulose and hemicellulose as response variables, running models including the two-way interactions among herbivore species, period, and grass content. We selected the models using AIC, retaining the model with the lowest number of parameters among the models within 2 units of AIC values.

All statistical analyses were performed in R for windows version 2.9.1 (R Development Core Team 2009), using library ‘ade4’ [56] and ‘nortest’ [57].

## Results

### Variation in Diet Composition

**Figure 1:**
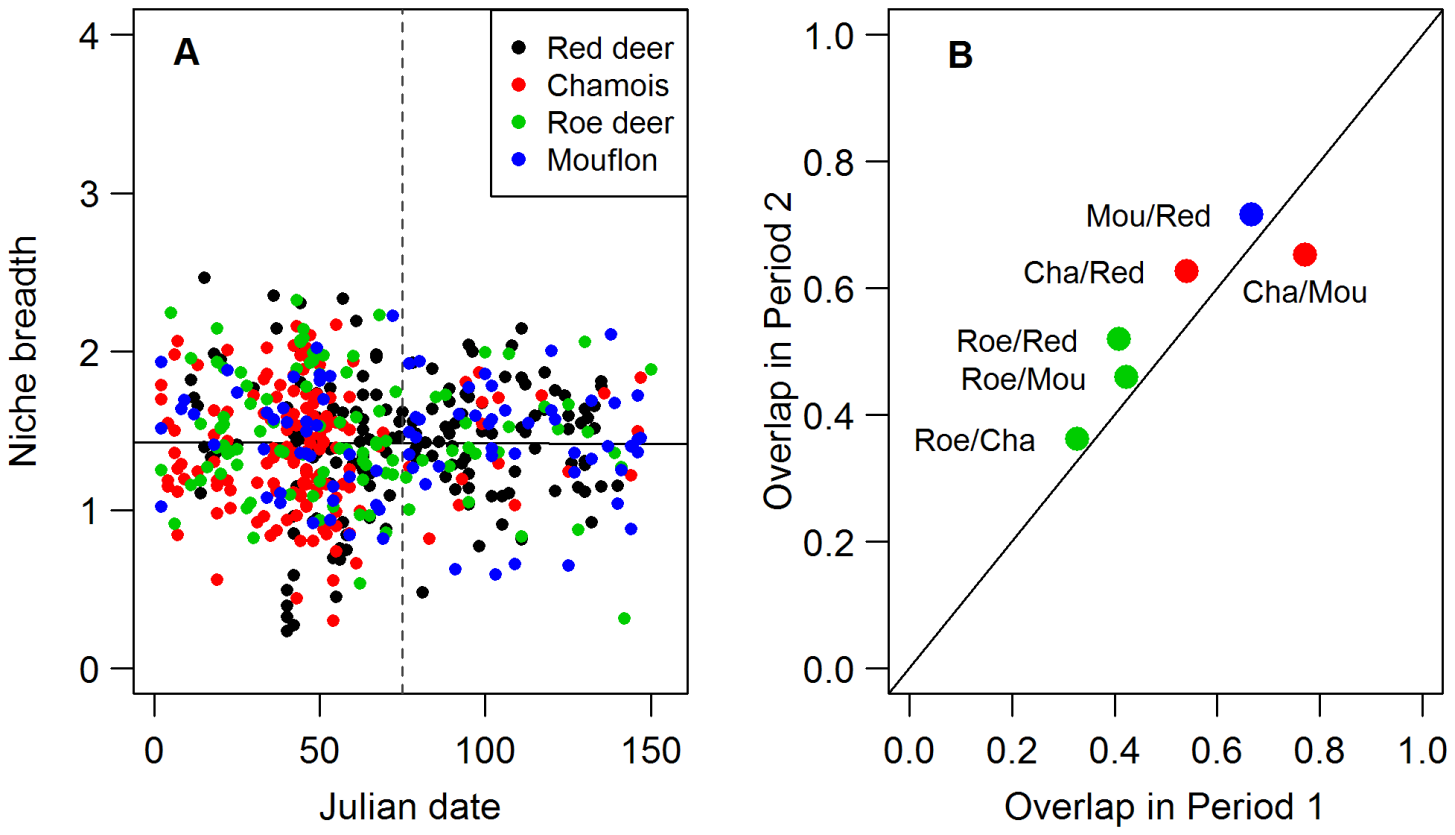
Niche breadth and overlap in diet among the four herbivore species. (A) Shannon-Wiener index of niche breadth according to julian date (1 being the 1^st^ of September). The predicted regression line is represented for all species combined. The dashed vertical line corresponds to the cutting date between periods 1 and 2 (15^th^ of November). (B) Schoener’s Index of overlap in periods 1 and 2. Schoener’s Index of overlap is given for the 6 combinations of species pairs and per period. The symbol colour is the colour corresponding to the smallest species in the pair, as used throughout the figures (green = roe deer, red = chamois, blue = mouflon, black = red deer ). The black line represents the expected values if there was no period effect.

Rumen contents were composed of 56 items or plant groups in roe deer, 60 in chamois, 68 in mouflon and 73 in red deer (details per period in Table S2 in File S1). Species diet niche breadth did not vary with date or species (interaction: F_3,487_ = 1.095, p = 0.351, main effect of species, F_3,487_ = 1.999, p = 0.309, main effect of date F_1,487_ = 0.439, p = 0.508, Fig. 1), thus giving no support that diet niche breadth should vary with rumen types.

Niche overlap was lowest for pairs involving roe deer and highest for chamois-mouflon and red deer-mouflon pairs (Fig. 1). The overlap did not increase from fall to winter (Wilcoxon rank tests: V = 14, p-value = 0.281, Fig. 1). Roe deer was overlapping the least with all other species in both periods, supporting the prediction that the species with a moose-type of rumen should show only a weak overlap with the species with an intermediate type of rumen. Among the latter species, overlap was important, with the highest overlap between chamois and mouflon in period 1 and between red deer and mouflon in period 2.

**Figure 2:**
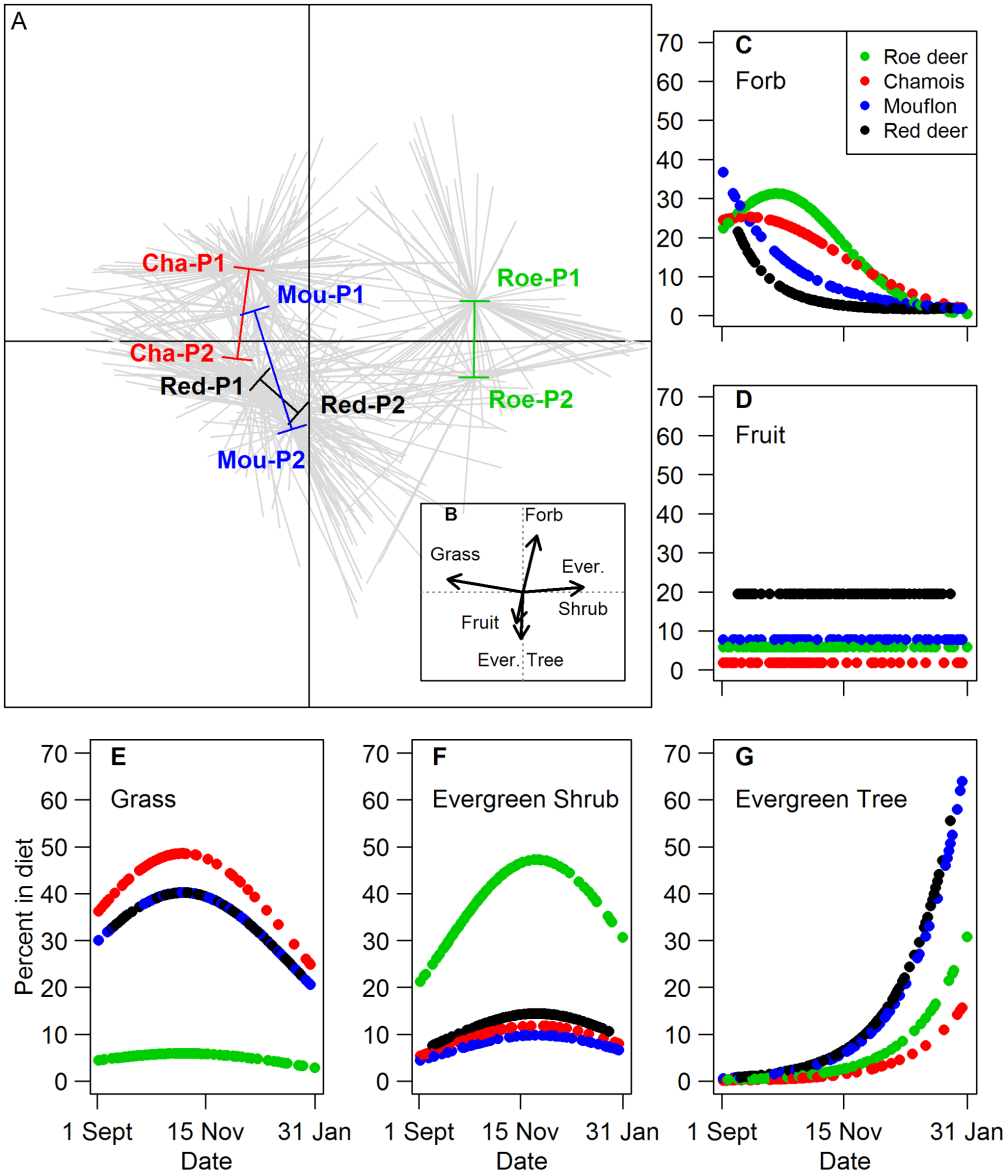
Patterns of diet composition according to species, periods and plant types. A. Position of herbivore species and periods on the two first axes of the between-PCA performed on the number of plant-type items per rumen. “P1” stands for Period 1 and “P2” for Period 2, “Cha” for Chamois, “Mou” for Mouflon, “Roe” for Roe deer, and “Red” for Red deer. Grey stars relate individual points for a given species and period to its gravity center. The shift in gravity centres from period 1 to period 2 is indicated by a coloured line for each species. B. Contribution of the first most important plant types to the axes of the between-PCA. “Ever. Tree” and “Ever. Shrub” are abbreviation for “Evergreen tree” and “Evergreen shrub” respectively. C to G. Variations in the percent of “Forb”, “Fruit”, “Grass”, “Evergreen shrub” and “Evergreen tree” respectively in the diet according to date for the four herbivore species as predicted from the best models.

The main plant types explaining the differences in diet composition within herbivore species and period were evergreen shrubs, grasses, forbs, evergreen trees and fruits (between-PCA test, *P* = 0.001). The first axis (63% of the total inertia) distinguished roe deer from the three other species on a gradient opposing evergreen shrubs (characterized by *Rubus fruticosus*) to grass (Fig. 2). The second axis (25% of total inertia) opposed forbs to evergreen trees/shrubs and fruits, mostly revealing the diet shift between periods towards less forbs and more evergreen trees (Fig. 2). Red deer was unique by having a high content of fruits (mainly apple and, to a lesser extent, acorn, Table S2 in File S1) and evergreen trees in both periods. The mouflon had the most pronounced temporal shift. In agreement with the values of overlap, its diet was relatively similar to that of chamois in period 1 (high grass and forbs contents) and to red deer in period 2 (high evergreen tree content, Fig. 2). Considering exclusively the 5 main plant types, species and date explained most of the variation in diet composition (except for fruits that showed no variation with date). The effect of date was non-linear for each plant type, except for fruits (Table 2, Fig. 2). From 1^st^ September to 14^th^ November, all herbivores increased their consumption of evergreen shrubs with time. From the 15^th^ November, the diet changed drastically, with a strong increase of evergreen tree consumption and a decrease in grass content (except in roe deer that hardly ever consumed grass). The interaction between date and species was selected for forbs only, as the decrease in forbs content with date occurred about one month later for roe deer and chamois than for red deer and mouflon (Fig. 2).

**Table 2:** Models on diet composition testing for effects of herbivores species and date.

Model on diet composition (plant types)		Grass	Evergreen shrub	Forb	Evergreen tree	Fruit
	df	ΔAIC	ΔAIC	ΔAIC	ΔAIC	ΔAIC
M1: Date	7	191.719	53.834	84.204	11.737	47.422
M2: Date+Date^2^	8	187.578	52.046	85.955	13.725	39.332
M3: Species	9	4.300	13.310	119.705	103.634	0
M4: Date+Species	10	3.727	6.459	15.773	0	1.669
M5: Date * Species	13	2.249	10.954	13.411	1.572	4.713
M6: Date * Species+Date^2^	14	0.529	4.423	10.496	2.596	3.687
M7: Date+Date^2^+Species	11	0	0	14.765	1.046	2.062
M8: Date * Species+Date^2^ * Species	17	4.968	3.453	0	3.562	3.179
BM1: Best model using 2 body mass classes	a	185.719	43.962	11.12	9.304	1.024
BM2: Best model using 3 morphophysiology categories	b	0.173	−1.281	5.341	10.705	38.814
BM3: Best model using 2 morphophysiology categories	c	−0.812	−1.792	7.094	11.708	42.859

Table gives the degrees of freedoms (df) and the delta-AIC (ΔAIC) between the best model and the specified model (M1 to M8, BM1 to BM3) for each plant type. The best model among the 8 first models denoted M1 to M8 has a delta-AIC of 0. The three last models, denoted BM1 to BM3, correspond to models replacing the herbivore species effect by herbivore species body mass categories (BM1) or categories based on digestive morphophysiology (BM2 and BM3). These models can be considered as better than the M1 to M8 models if their delta-AIC is negative.

a : Number of degrees of freedom of the best model minus 2.

b : Number of degrees of freedom of the best model minus 1.

c : Number of degrees of freedom of the best model minus 2.

The classification opposing roe deer to the other 3 species accounted for the species effect in terms of grass and evergreen shrubs contents in the diet. Mouflon did not differ from chamois and red deer for these plant types. Body mass categories (large *vs* small) accounted for species differences in terms of fruit content (only red deer, the largest species, ate a relatively large amount of fruits, Table 2). For the three other plant types, the effect of herbivore species could not be accounted for by body mass or rumen type groupings.

### Variation in Diet Quality

**Figure 3:**
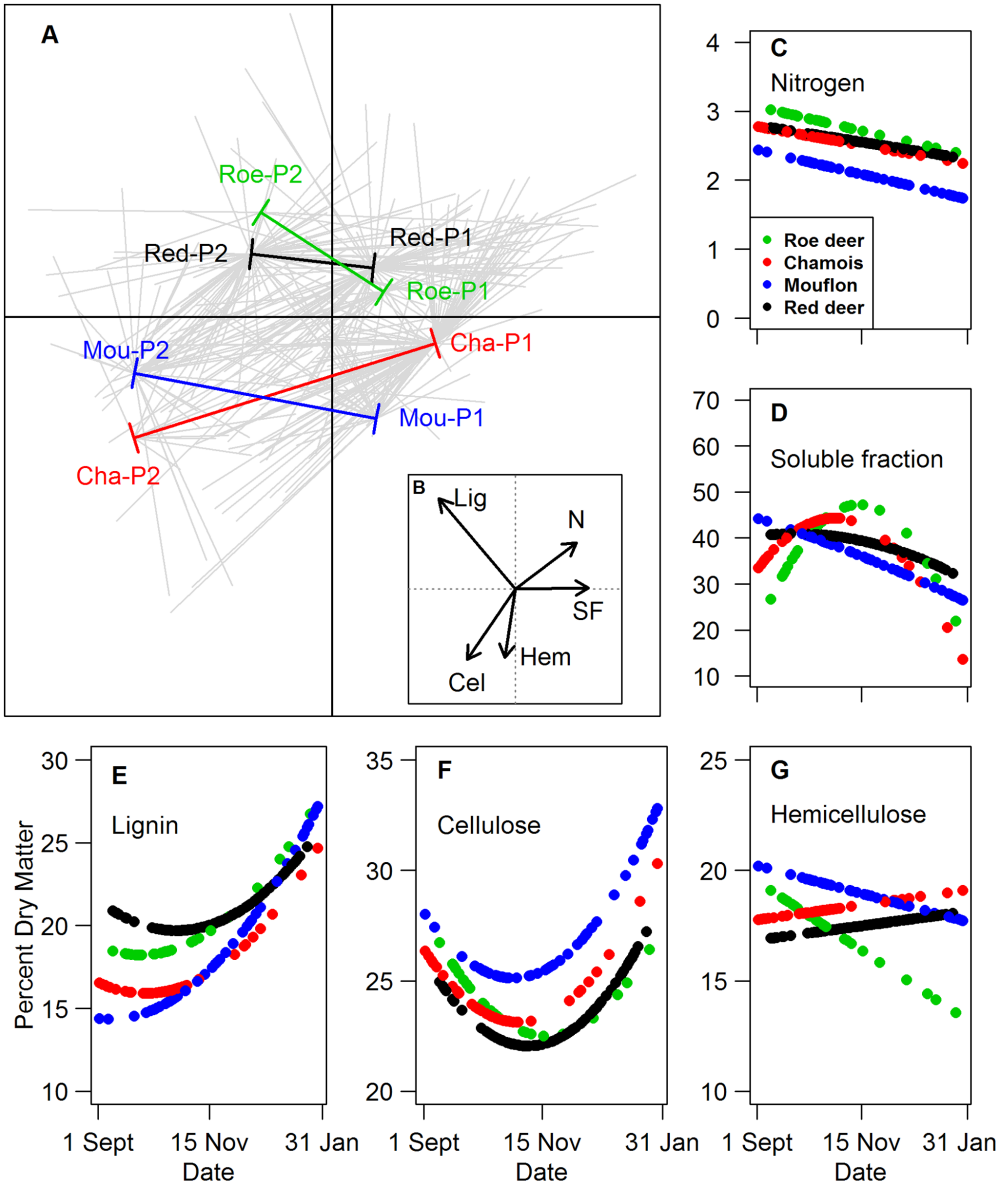
Patterns of diet quality according to species, periods and nutrients. A. Position of herbivore species and period on the two first axes of the between-PCA performed on the analyses of rumen nutrient content. “P1” stands for Period 1 and “P2” for Period 2, “Cha” for Chamois, “Mou” for Mouflon, “Roe” for Roe deer, and “Red” for Red deer. Grey stars relate individual points for a given species and period to its gravity center. The shift in gravity centres from period 1 to period 2 is indicated by a coloured line for each species. B. Contribution of each chemical component to the between PCA. “N” stands for “Nitrogen”, “SF” for “Soluble Fraction”, “Lig” for “Lignine”, “Cel” for “Cellulose”, and “Hem” for “Hemicellulose”. C to G. Variations in the percent of “Nitrogen”, “Soluble Fraction” “Lignine”, “Cel” for “Cellulose”, and “Hem” for “Hemicellulose” respectively dry matter according to date for the four herbivore species as predicted from the best models.

Diet quality varied mainly with period and to a lesser extent with the herbivore species (Fig. 3). Diets with high nitrogen contents also had high soluble fraction and low amount of cellulose. This covariation mainly contributed to the first axis of the PCA. Lignin content in the diet varied independently of the content of nitrogen, soluble fraction, and cellulose. The period effect is displayed mainly by a shift on the first axis (64% of the total inertia) towards lower nitrogen and soluble fractions for all species. The second axis (25% of the total inertia) accounted mainly for species differences in content of hemicellulose and lignin (Fig. 3). Red deer and roe deer were close in both periods, indicating that large body size was not correlated with a low nitrogen content.

Models performed on each measure of diet quality confirmed the strong decrease of quality with date for all herbivore species (drop in nitrogen content coupled to an increase in the poorly digestible lignin). The effect of date was linear for nitrogen and hemicellulose and quadratic for lignin, soluble fraction and cellulose. The date and species interaction was significant for lignin and soluble fraction (Table 3): soluble fraction decreased a month later in chamois and roe deer than in mouflon and red deer, while lignin increased more sharply for mouflon than for any other species (Fig. 3). Mouflon’s diet quality differed from all the 3 other species by its high cellulose and low nitrogen contents, despite its high overlap in terms of diet composition with chamois and red deer (see above). Chamois and red deer diet quality were relatively similar, but for the lignin content, which was higher in red deer (segregation on axis 2 of the PCA, Fig. 3).

**Table 3:** Models on diet quality (content in hemicellulose (Hem), lignin (Lig), cellulose (Cel), soluble fraction (SF) and nitrogen (N) per rumen) testing for the relationship between herbivores species and date.

Modelon diet quality		Lignine	Hemicellulose	Nitrogen	Cellulose	Soluble Fraction
	df	ΔAIC	ΔAIC	ΔAIC	ΔAIC	ΔAIC
M1: Date	4	19.504	7.597	64.8016	30.612	7.375
M2: Date+Date^2^	5	20.301	8.296	66.7746	25.704	7.796
M3: Species	6	69.885	1.364	49.3135	24.999	56.439
M4: Date+Species	7	6.76	3.026	0.7433	1.443	3.391
M5: Date * Species	10	1.4	0	0	4.843	2.294
M6: Date * Species+Date^2^	11	0	0.794	1.9034	3.173	2.144
M7: Date+Date^2^+Species	8	4.949	2.505	1.7678	0	4.187
M8: Date * Species+Date^2^ * Species	14	1.969	2.64	2.2158	2.797	0
BM1: Best model using 2 body mass classes	a	−3.878	7.504	50.2496	9.035	4.859
BM2: Best model using 3 morphophysiology categories	b	20.354	2.182	4.9709	6.996	0.724
BM3: Best model using 2 morphophysiology categories	c	24.145	4.91	37.1155	25.228	5.399

Then, effect of species in the best model, is replaced by an alternative effect (body mass, diet category and breeder ability). The selected model (i.e. with the lowest AIC value) is in shaded cells.

Table gives the degrees of freedoms (df) and the delta-AIC (ΔAIC) between the best model and the specified model (M1 to M8, BM1 to BM3) for each chemical component. The best model among the 8 first models denoted M1 to M8 has a delta-AIC of 0. The three last models, denoted BM1 to BM3, correspond to models replacing the herbivore species effect by herbivore species body mass categories (BM1) or categories based on digestive morphophysiology (BM2 and BM3). These models can be considered as better than the M1 to M8 model if their delta-AIC is negative.

a : Number of degrees of freedom of the best model minus 2.

b : Number of degrees of freedom of the best model minus 1.

c : Number of degrees of freedom of the best model minus 2.

Inter-specific differences in lignin content were well accounted for by the body mass category, supporting that the largest species ate a more lignified diet while inter-specific differences in soluble fraction were best explained by the 3 categories opposing roe deer, chamois and red deer pooled together, and mouflon (Table 3). For the three other diet quality proxies, the effect of herbivore species could not be accounted for by any of the predefined grouping (body mass or the 2 groupings based on rumen types), mainly because of the outstanding position of the mouflon in terms of lignin and cellulose contents.

### Relationships between Diet Composition and Diet Quality

Composition in terms of plant types and diet quality were correlated for both periods and all species except for mouflon in period 1 and for roe deer, for which the sample size was very low (Table 4). The coefficient of coinertia (RV) increased strongly from period 1 to period 2 for all species, except for red deer, with a marked gap between the three smallest species (all RVs>35%) and the largest one, red deer (RV = 9%). The highest relative values for the three smallest species compared to red deer exemplifies that these species relied on fewer plant types to maintain high nitrogen- and high soluble fractions in their diet during the food-restricted period. This does not support that species with an intermediate type of rumen should have the lowest correlation between composition and quality, as chamois and mouflon markedly differed from red deer.

**Table 4:** Coefficient of coinertia between diet composition in terms of plant types and diet quality, per species and period.

Species	Period 1			Period 2		
	RV	N	P	RV	N	P
Roe deer	13%	22	0.233	53%	6	0.105
Chamois	12%	90	0.002	47%	10	0.057
Mouflon	15%	30	0.102	35%	34	<0.001
Red deer	10%	64	0.029	9%	66	0.026

Table gives coefficient of inertia (RV), sample size (N) and significance values obtained by bootstrapping (P) for period 1 (September to mid November) and period 2 (mid-November to January) for the 4 herbivore species.

Interestingly, grasses were correlated with high quality diets (in terms of nitrogen and soluble fraction) for red deer, mouflon and chamois, though this covariation disappeared in period 2 for the two latter species (Figure S3 in File S1). Evergreen shrubs played a determinant role for the diet quality in all species, but this was most pronounced for chamois and roe deer (the two smallest species), and for mouflon in period 2. The decrease in diet quality, i.e. related to increasing contents of lignin and cellulose from period 1 to period 2, corresponded to increasing consumption of evergreen trees for all species (Figure S3 in File S1). While evergreen shrubs can explain the content of high nitrogen and cellular tissue in chamois, roe deer and mouflon in period 1, the actual plant species used within this plant type differed, supporting that not all herbivore species rely on the same ‘key-plants’. Mouflon in period 1 and chamois browsed mainly on *Actrostaphylos uva-ursi* and *Ligustrum vulgare,* while roe deer mainly consumed *Hedera helix* and *Rubus fructicosus*. Similarly, while fruits played a major role in explaining the high quality diet of red deer and mouflon, red deer consumed a higher fruit diversity than mouflon (Table S2 in File S1). Evergreen trees, which constituted a greater part of the diet for all four species in period 2 (Fig. 2) never co-occurred with proxies of high quality diet (it correlated with high lignin content) and as such, might not be considered as ‘key-plants’ for any of the herbivore species.

**Figure 4:**
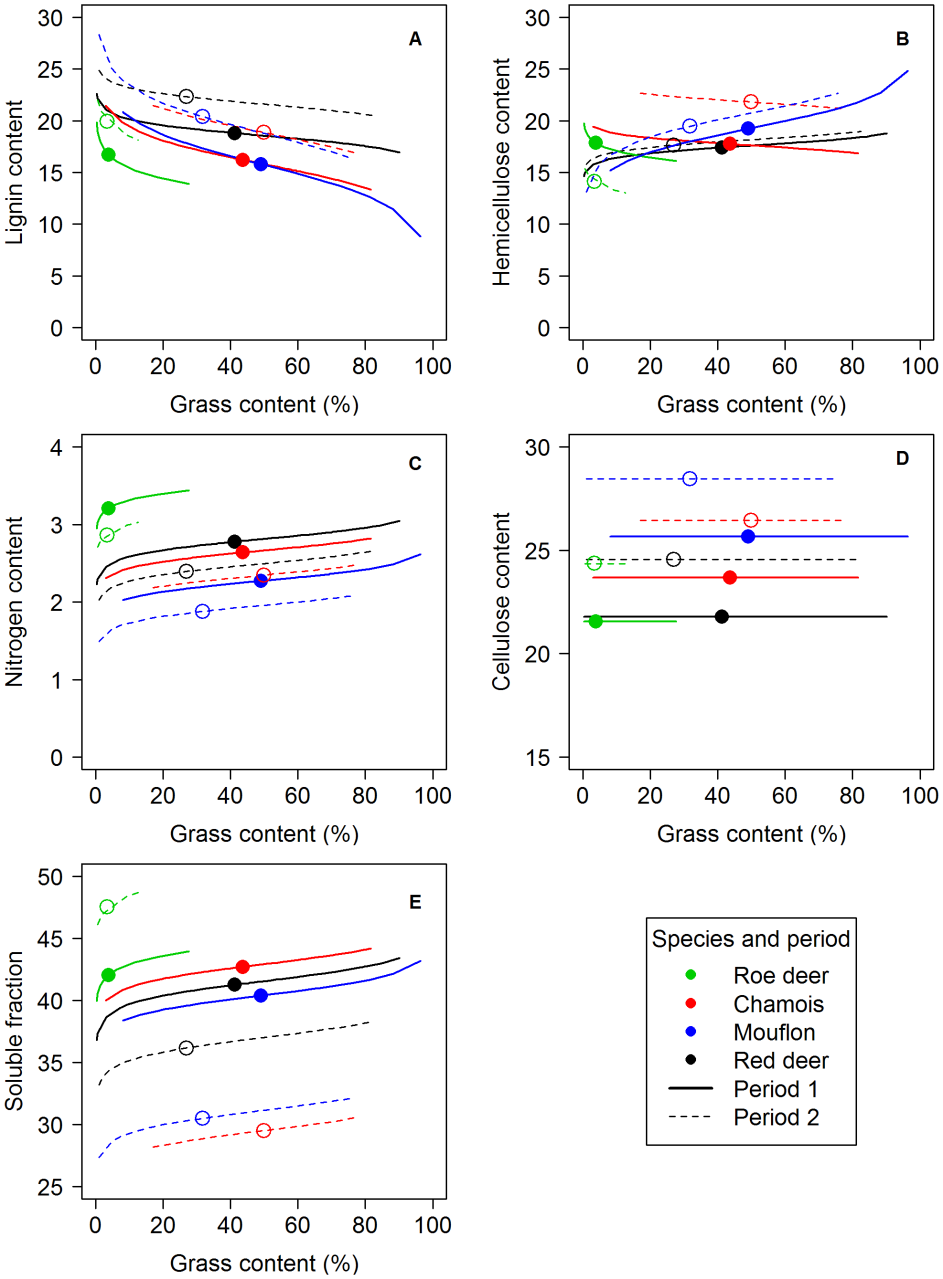
Relationship between the percentage of grass content in the rumen and diet quality. A–E. Expected values of respectively “Nitrogen”, “Helicellulose”, “Lignin”, “Cellulose”, and “Soluble Fraction” according to the grass content in the rumen, species and period. Average values of grass content per herbivore species and period and their corresponding predicted value are added to help visualising the gravity centres of the actual data in terms of both grass content and chemical component.

When testing whether diet quality proxies (nitrogen, lignin, cellulose and hemicellulose contents) were well predicted by the percentage of grass in the diet, we found significant differences among species in terms of intercept (main species effect, Fig. 4, list of models in Table S3 in File S1). The interaction between grass content and species effect was clearly significant for lignin and hemicellulose contents (Table S3 in File S1). For lignin, this interaction was explained by a shallower decrease with increasing grass content for red deer (b = −0.799±0.260) than for chamois, mouflon and roe deer (chamois b = −1.682±0.511; mouflon: b = −2.269±0.421; roe deer: b = −1.331±0.813; Fig. 4). For hemicellulose, patterns were more complex (Fig. 4), with a non-significant decrease of hemicellulose content with increasing grass content for roe deer (b = −0.762±0.836) and chamois (b = −0.583±0.529), and a significant increase in the cases of mouflon (b = 0.799±0.260) and red deer (b = 0.493±0.270).

Nitrogen level had a positive relationship with grass content (b = 0.120±0.003) which was similar for all species and in both periods (Fig. 4). Likewise, soluble fraction increased with grass content for all species alike in both periods (b = 0.120±0.003). Differences among species were marginal in period 1 but large in period 2 (Fig. 4), as chamois and mouflon had lower soluble fraction values than red deer, and roe deer had the highest values of all. In contrast, cellulose, while being species-specific and period-specific (Fig. 4) did not vary with grass content in the diet.

## Discussion

By studying the diet composition and quality of 4 ungulate species coexisting on the same mountain range, we found that (1) despite large changes in diet composition from fall to winter, diet breadth remained similar for all species, while patterns of diet overlap among species depended first and foremost on differences in rumen types (high among species of intermediate rumen type, low between those and roe deer, a species with a “moose-type” rumen); (2) roe deer, the species with a “moose-type” rumen, was confirmed as a browser and especially, an “obligatory non-grazer”, while mouflon was classified with chamois and red deer as an intermediate feeder and not as a grazer, in agreement with a recent review ([33]); (3) the relationship between diet composition and quality differed among herbivore species, with the most remarkable feature being that diet quality proxies for mouflon varied with the amount of grass content in its diet in a different way than for the other species, and (4) the actual plant species used during winter, which determined the diet quality was herbivore species-specific, suggesting that the concept of ‘key-resources’ (*sensu* [24]) may mostly apply to the smallest “moose-type” species, the roe deer.

### Body Mass and Digestive Morphophysiology as Covariates Explaining Diet Composition and Quality

Diet composition and diet quality differed among species in subtle and complex ways: species-specific differences were indeed not consistent for all plant types and all diet quality proxies. The clearest pattern was the corroboration that rumen type constrains diet composition, as the “moose-type” species (roe deer) was indeed an “obligate non-grazer” [6], [21] from fall to winter. The outlying position of this species also stood out through the constantly higher nitrogen content of rumen samples compared to the three other species (Fig. 3 and 4), as expected from a species feeding mainly from browse material [18], [19], [27], [58]. Browse has been shown to contain both a high level of soluble sugars and proteins, and a high level of tannins and lignin, the two latter preventing nutrients to be easily digested [17]. The mechanical properties of grass and browse in the rumen [59], and the ability to consume tannin-rich forage may constrain species-specific diet’s plasticity [34], [20]. Our results support a lower plasticity in the “moose-type” species resulting in grass avoidance.

On the opposite, chamois, mouflon and red deer had a higher overlap in terms of diet composition, consuming both grass and dicotyledons in large quantities, as expected from species with intermediate type of rumen. From its diet composition, mouflon was very similar to chamois and red deer [16], [60], but it shifted food to a greater extend between fall and winter than chamois and red deer. Mouflon had indeed a flexible diet (reviewed in [33]), a pattern differing from [27] in a study site where mouflon avoided woody plants even in winter. It shows that rumen type was a good predictor of food assemblages in the diet in terms of rough categories (grass, dicotyledons, fruits, see [15]), but cannot, alone, predict temporal patterns in diet composition and overlap. In addition, two striking patterns separated mouflon from the two species with intermediate rumen types: despite similarities in composition, its diet was lower in nitrogen and higher in cellulose and hemicellulose contents than that of red deer or chamois. Accordingly, mouflon was outstanding both in terms of diet quality and in terms of the slope of the relationship between grass content and diet quality variables, patterns that cannot be accounted for by its body mass or rumen type. This may reflect the use of different parts of plant or different grass species by mouflon.

In support to the prediction that a large body mass should covary with a diet of lower quality, the largest species, red deer, had a diet with a high content of lignin. In addition, red deer stood out by its high consumption of large fruits, a high energetic food [61], often used by cervids [54], [61]. The red deer largest mouth size compared to the three other species may contribute to its relatively more intense use of apple and chestnut, not found in other species.

### Species-specific Pathways to Maintain a High Quality Diet during Plant Dormancy

The four species suffered from a general decrease in diet quality with a drop in nitrogen and soluble fraction coupled to an increase of poorly digestible components such as lignin. However, they coped differently with this loss of food availability and quality. Indeed, the strength of the quality to composition relationship got reinforced in the winter for all species, excepted for red deer, suggesting that roe deer, chamois and mouflon rely (in terms of diet quality) on a restricted number of items while red deer may use a larger array resources for maintaining the same diet quality. The notion of ‘key-resources’ has been developed by Illius and O’Connor [24] who stipulated that population dynamics may depend on few resources on which population ‘key-factors’ may depend. To identify ‘key-resources’ when demographic data are unavailable, a preliminary step is to unravel, as we did here, the relationship between diet composition and diet quality. In our study sites, most natural adult mortality occurs during or at the end of winter, and may therefore depend partly on the access to ‘key-resources’ during this period [62]. The four herbivores species were heavily relying on evergreen trees and shrubs at the end of the autumn and start of the winter (particularly *Rubus fruticosus, Actostaphylos uva-ursi, Hedera helix,* acorn, *Abies alba* and *Picea abies*) which could be ‘key-plants’ [26]. *Rubus fruticosus* and *Abies alba* clearly appeared to be simultaneously consumed by the four species of herbivores. However, the correlation between diet composition and diet quality was low for red deer, suggesting that different types of diet composition led to similar values of diet quality. In these circumstances, it may be more appropriate for red deer to investigate the concept of ‘key-menus’, trying to understand which particular quality criteria red deer may be seeking when assembling a diverse diet (*e.g.* [18]). We therefore posit here that the concept of ‘key-resources’ may not easily apply for all herbivores as species-specific diet selection and ability to select specific plant parts may blur the quality to composition relationship. This concept of ‘key-resources’ may apply better to the most selective species, most probably towards small body mass species and the “moose-type” digestive morphophysiology. While we lacked power in terms of sample size to really test whether the correlation between diet composition and diet quality was highest for the “moose type” (roe deer), our results nevertheless indicated that it could be the case, which should be tested in more diverse communities.

### Insights for Possible Competitive Relationships among Mountain Herbivore Communities

Niche breadth often decreases during period of plant dormancy as a result to a sharp decline in the number of plants available (*e.g*. [63], [27]). This was not supported by our results, even though snow covers the ground from mid-November and contributes even more to the decrease of plant availability, This suggests that all herbivore species became less selective during winter (a larger proportion of plants available was used) and had to resort to non-preferred food items (*e.g* [48]). Interestingly, this did not lead to an increased overlap among species from fall to winter. Possible explanations when large overlaps are observed are (1) non limiting resources, (2) segregation occurring at a higher spatial level, rather than on the trophic axes of the niche, and (3) segregation occurring within plant species,. The large vegetation and landscape heterogeneity at small spatial scale found in mountainous environment should certainly favour species coexistence (*e.g.* [63], [64], [65]). For instance, the latter may explain why roe deer diet did not overlap to a great extent with red deer diet, in contrast to several previous findings [44], [28], [30], [38] though both are forest-dwelling species and overlapping in geographic range in our study area (Figure S1 in File S1). However, our study clearly shows that there is a potential for competition among these species, and that, with the colonisation by roe and red deer still on-going [59], we may see different patterns of overlap in diet in the future, which could lead to an increase in exploitation competition and lead to trophic niche segregation.

## Conclusion

The diet niche of a species is dynamic and depends on joint processes of forage selection and resource availability (*e.g.* [55], [18], [24], [48]). Our study, investigating whether diet’s patterns (in terms of composition and quality) and changes in diet agree with prediction from body mass or rumen type, shows that diet assemblage is a complex process, and that this complexity in how large herbivore exploit and share food resources needs to be interpreted in the light of other ecological or behavioural echanisms such as space use at a larger scale or plant organ selection at the bite scale. Our results concur with [66] who suggested that species differences in terms of diet may be less pronounced in European herbivore communities than in African ones. The approach we undertook, by analysing species-specific relationship between diet composition and diet quality, should however be tested in more diverse communities, such as in Africa, before concluding that distinction in diets is more blurred in Europe than in Africa. It also points out towards several lines for future research concerning methodological, morphophysiological and ecological aspects. For instance, as pointed out by [27] or [48], results on diet breadth and overlap have to be interpreted with caution, given that studies based on either microhistology (in the cases of feces analyses) or identification through microscope (as with gut samples) do not allow determining all plant eaten to the species level. This can lead to an underestimation of the number of species, with a more pronounced effect for some plants groups (forbs and grasses) than for others. Resorting to more precise identification, such as DNA-barcoding [67], [68], would be a helpful way to reevalute such results (*e.g.* [69]). We have also underlined the need for a better understanding of which plant parts are selected by each herbivore species, as this may contribute to explain why the diet composition to diet quality relationship can be species-specific [34]. This is fundamental to better understand the connection between diet assemblages and the energy acquired by individuals and ultimately, demographic performances. Finally, the impact of a large community of herbivores, diverse in its patterns of food resource use, would need to be explored further, because it can impact both primary and secondary production to an extent that has been little evaluated so far [70]. This is particularly true in mountains, where changes in temperature and land use occur rapidly [68], and where the role of large herbivores on landscape dynamics and the maintenance of biodiversity may become large now that the populations of all species have increased in numbers and distribution [34].

## Supporting Information

File S1 **This file contains Figure S1–S3 and Table S1–S3.** Figure S1, Distribution of roe deer, chamois, mouflon and red deer on our study area. Ninety-five percent and 50 percent kernel distribution ranges are displayed. Data used to calculate these distribution are the location of all harvested individuals during the study period, i.e. from 2003 to 2008. The background map in shades of grey correspond to altitude gradient (the lighter the higher). Figure S2, Altitudinal distribution of the four species in our study area. All animals harvested from 2003 to 2008 were pooled to calculate the distribution across altitudes. Figure S3, Relationship between diet’s composition and quality for the two periods performed using co-inertia analysis on two PCA (abundance per plant type in rumen and chemical content as hemicellulose (Hem), lignin (Lig), cellulose (Cel), soluble fraction (SF) and nitrogen (N) in rumen). The 3 chemical components and the plant types contributing the most to the axes are displayed as arrows allowing axes interpretation. Absolute lengths of the arrows are arbitrary and chosen to display well on the figure. Table S1, Composition of the rumen data set for diet composition. Table S2, Diet composition in percentage according to the two periods of limiting season (period 1: 1st September to 15th November; periods 2: 16th November to 31st January). Table S3, Best models for the analyses of the relationship between grass content in the diet and lignin, hemicellulose, nitrogen, cellulose and soluble fraction (sol. fraction) contents. Models with AIC within 2 units of the model with the lowest AIC are presented with their number of parameters, DAIC (difference with the best model), and AIC weight. Among the models with close AIC values, we selected the model with the lowest number of parameters. Figures with the predicted values is in the main body of the text (Fig. 5).
(DOC)
