# Supplementary material for: Intra- and Interspecific Differences in Diet Quality and Composition in a Large Herbivore Community
Source: PLoS One. 2014 Feb 24;9(2):e84756. doi: 10.1371/journal.pone.0084756 (PMC3933327; doi:10.1371/journal.pone.0084756)
Supplement: File S1 — This file contains Figure S1–S3 and Table S1–S3. Figure S1, Distribution of roe deer, chamois, mouflon and red deer on our study area. Ninety-five percent and 50 percent kernel distribution ranges are displayed. Data used to calculate these distribution are the location of all harvested individuals during the study period, i.e. from 2003 to 2008. The background map in shades of grey correspond to altitude gradient (the lighter the higher). Figure S2, Altitudinal distribution of the four species in our study area. All animals harvested from 2003 to 2008 were pooled to calculate the distribution across altitudes. Figure S3, Relationship between diet’s composition and quality for the two periods performed using co-inertia analysis on two PCA (abundance per plant type in rumen and chemical content as hemicellulose (Hem), lignin (Lig), cellulose (Cel), soluble fraction (SF) and nitrogen (N) in rumen). The 3 chemical components and the plant types contributing the most to the axes are displayed as arrows allowing axes interpretation. Absolute lengths of the arrows are arbitrary and chosen to display well on the figure. Table S1, Composition of the rumen data set for diet composition. Table S2, Diet composition in percentage according to the two periods of limiting season (period 1: 1st September to 15th November; periods 2: 16th November to 31st January). Table S3, Best models for the analyses of the relationship between grass content in the diet and lignin, hemicellulose, nitrogen, cellulose and soluble fraction (sol. fraction) contents. Models with AIC within 2 units of the model with the lowest AIC are presented with their number of parameters, DAIC (difference with the best model), and AIC weight. Among the models with close AIC values, we selected the model with the lowest number of parameters. Figures with the predicted values is in the main body of the text (Fig. 5). (DOC) [file pone.0084756.s001.doc]

**Figure S1**. Distribution of roe deer, chamois, mouflon and red deer on our study area. Ninety-five percent and 50 percent kernel distribution ranges are displayed. Data used to calculate these distribution are the location of all harvested individuals during the study period, i.e. from 2003 to 2008. The background map in shades of grey correspond to altitude gradient (the lighter the higher).


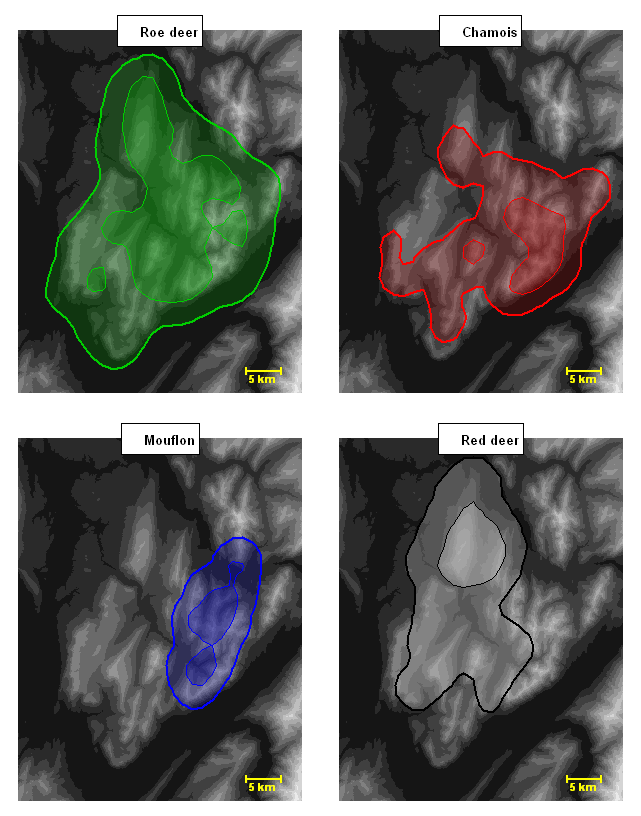


**Figure S2.** Altitudinal distribution of the four species in our study area. All animals harvested from 2003 to 2008 were pooled to calculate the distribution across altitudes.


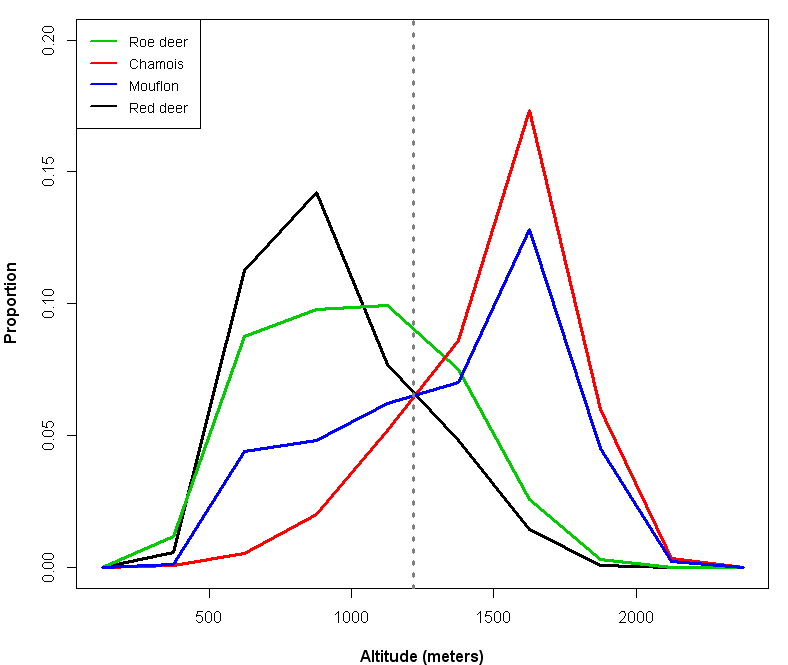


**Table S1.** Composition of the rumen data set for diet composition.

|  | **Roe deer** | **Chamois** | **Mouflon** | **Red deer** |
| --- | --- | --- | --- | --- |
| **Sex ratio** |  |  |  |  |
| male | 53 | 71 | 47 | 92 |
| female | 51 | 77 | 38 | 66 |
| unknown | - | - | 1 | - |
| **Age** |  |  |  |  |
| adult | 97 | 92 | 60 | 102 |
| sub-adult | - | 37 | 14 | 42 |
| young | 7 | 19 | 12 | 14 |

**Table S2.** Diet composition in percentage according to the two periods of limiting season (period 1: 1^st^ September to 15^th^ November; periods 2: 16^th^ November to 31^st^ January)

| Growth form | Item | | **Chamois** | | | **Mouflon** | | | **Red deer** | | | **Roe deer** | | |
| --- | --- | --- | --- | --- | --- | --- | --- | --- | --- | --- | --- | --- | --- | --- |
|  |  |  | P1 | P2 | Total | P1 | P2 | Total | P1 | P2 | Total | P1 | P2 | Total |
| **Fruit** |  | | **1.29** | **1.48** | **1.31** | **5.18** | **4.43** | **4.79** | **19.54** | **12.45** | **16.18** | **3.44** | **5.83** | **4.09** |
|  | *Alnus.viridis* | | - | 0.35 | 0.04 | - | - | - | - | 0.02 | 0.01 | - | - | - |
|  | *Castanea.sativa* | | - | - | - | - | - | - | 1.42 | 2.59 | 1.97 | - | - | - |
|  | *Chaerophyllum.temulentum* | | 0.10 | - | 0.09 | - | - | - | - | - | - | - | - | - |
|  | *Corylus.avellana* | | - | - | - | - | 0.17 | 0.09 | - | 0.01 | 0.01 | 0.01 | 0.04 | 0.02 |
|  | *Fagus.sylvatica* | | - | - | - | - | 0.04 | 0.02 | 0.49 | 0.70 | 0.59 | - | - | - |
|  | *Heraclum.sphondylium* | | 0.11 | - | 0.10 | - | - | - | - | - | - | - | - | - |
|  | *Lonicera.xylosteum* | | 0.12 | - | 0.11 | - | - | - | - | - | - | - | - | - |
|  | *Malus.sylvestris* | | - | 1.13 | 0.12 | - | 0.05 | 0.03 | 10.20 | 7.29 | 8.82 | 1.54 | 2.05 | 1.68 |
|  | *Onobrychis.viciifolia* | | 0.03 | - | 0.03 | - | - | - | - | - | - | - | - | - |
|  | *Pinus.sylvestris* | | - | - | - | - | - | - | - | 0.27 | 0.13 | - | - | - |
|  | *Pyrus.pyraster* | | - | - | - | - | - | - | 1.64 | 0.58 | 1.14 | - | - | - |
|  | *Quercus.sp* | | - | - | - | 4.73 | 4.16 | 4.43 | 4.10 | 0.83 | 2.54 | 1.43 | 2.99 | 1.85 |
|  | *Rosa.sp* | | 0.92 | - | 0.82 | 0.45 | - | 0.21 | 0.95 | 0.04 | 0.52 | 0.46 | - | 0.34 |
|  | *Sorbus.aria* | | - | - | - | - | - | - | 0.33 | - | 0.17 | - | - | - |
|  | *Sorbus.aucuparia* | | - | - | - | - | - | - | 0.01 | - | 0.01 | - | - | - |
|  | *Zea.mays* | | - | - | - | - | - | - | 0.40 | 0.13 | 0.27 | - | 0.76 | 0.21 |
| **Fern** |  | | **-** | **-** | **-** | **-** | **2.36** | **1.23** | **0.06** | **-** | **0.03** | **0.02** | **0.19** | **0.07** |
|  | *Asplenium.viride* | | - | - | - | - | - | - | 0.03 | - | 0.01 | - | - | - |
|  | *Dryopteris.sp* | | - | - | - | - | 0.39 | 0.20 | - | - | - | - | 0.13 | 0.04 |
|  | *Lycopodium.sp* | | - | - | - | - | 0.02 | 0.01 | - | - | - | - | - | - |
|  | *Other.fern* | | - | - | - | - | 1.21 | 0.63 | 0.03 | - | 0.02 | 0.02 | 0.04 | 0.03 |
|  | *Polystichum.aculeatum* | | - | - | - | - | 0.74 | 0.39 | - | - | - | - | 0.02 | 0.01 |
| **Grass** |  | | **44.44** | **50.46** | **45.09** | **41.78** | **29.03** | **35.11** | **39.76** | **33.37** | **36.73** | **5.08** | **5.52** | **5.20** |
|  | *Graminoids* | | 44.44 | 50.46 | 45.09 | 41.78 | 28.94 | 35.06 | 38.99 | 32.32 | 35.82 | 5.08 | 5.52 | 5.20 |
|  | *Zea.mays* | | - | - | - | - | 0.09 | 0.05 | 0.77 | 1.05 | 0.91 | - | - | - |
| **Legume** | | | **2.61** | **-** | **2.33** | **0.05** | **-** | **0.02** | **0.29** | **0.02** | **0.16** | **0.56** | **0.07** | **0.43** |
|  | *Coronilla.emerus* | | - | - | - | - | - | - | - | - | - | - | 0,01 | < 0,006 |
|  | *Coronilla.minima* | | 0.06 | - | 0.05 | - | - | - | - | - | - | 0,01 | - | 0,01 |
|  | *Onobrychis.viciifolia* | | 0.01 | - | 0.01 | - | - | - | - | - | - | - | - | - |
|  | *Trifolium.medium* | | 0.40 | - | 0.35 | - | - | - | - | - | - | - | - | - |
|  | *Trifolium.sp* | | 2.14 | - | 1.91 | 0.05 | - | 0,02 | 0,29 | 0,02 | 0,16 | 0,55 | 0,06 | 0,42 |
| **Forb** |  | | **22.43** | **9.00** | **20.98** | **15.69** | **3.71** | **9,42** | **6,17** | **1,84** | **4,11** | **25,07** | **10,37** | **21,11** |
|  | *Aconitum.sp* | | < 0.006 | - | < 0.006 | - | - | - | - | - | - | - | - | - |
|  | *Alchemilla.alpina* | | - | - | - | - | 0.10 | 0,05 | - | - | - | - | - | - |
|  | *Alchemilla.xanthochlora* | | - | - | - | - | - | - | - | - | - | 0,23 | - | 0,17 |
|  | *Epilobium.sp* | | 0.03 | - | 0.02 | - | - | - | - | - | - | - | - | - |
|  | *Galium.mollugo* | | 0.01 | - | 0.01 | - | - | - | - | - | - | - | - | - |
|  | *Galium.odoratum* | | 0.01 | - | 0.01 | - | - | - | 0,27 | 0,04 | 0,16 | 0,36 | - | 0,26 |
|  | *Gentiana.lutea* | | - | - | - | - | - | - | 0,03 | - | 0,02 | 0,38 | - | 0,28 |
|  | *Geranium.sylvaticum* | | 0.06 | - | 0.05 | 0.26 | - | 0,12 | - | - | - | 1,00 | 0,25 | 0,80 |
|  | *Geum.montanum* | | - | - | - | 0.01 | - | < 0,006 | - | - | - | - | - | - |
|  | *Hypericum.perforatum* | | 0.10 | - | 0.09 | 0.32 | - | 0,15 | 0,09 | - | 0,05 | 0,24 | 0,63 | 0,35 |
|  | *Lamium.galeonbdolon* | | - | - | - | - | - | - | 0,18 | - | 0,09 | - | 0,24 | 0,06 |
|  | *Other.forb* | | 17.91 | 6.67 | 16.69 | 14.54 | 3.39 | 8,71 | 5,41 | 1,69 | 3,64 | 20,82 | 8,12 | 17,40 |
|  | *Oxalis.acetosella* | | - | - | - | - | - | - | - | - | - | 0,06 | - | 0,04 |
|  | *Plantago.sp* | | 0.74 | 0.25 | 0.69 | 0.01 | - | < 0,006 | 0,05 | 0,04 | 0,05 | 0,08 | - | 0,06 |
|  | *Potentilla.sterilis* | | 0.02 | - | 0.02 | 0.02 | - | 0,01 | 0,06 | 0,01 | 0,03 | 0,39 | 0,49 | 0,42 |
|  | *Sanguisorba.minor* | | 0.03 | - | 0.02 | - | 0.05 | 0,03 | 0,08 | 0,06 | 0,07 | 0,70 | 0,64 | 0,69 |
|  | *Soldanella.alpina* | | < 0.006 | - | < 0.006 | 0.02 | - | 0,01 | - | - | - | - | - | - |
|  | *Stellaria.sp* | | 0.02 | - | 0.02 | - | - | - | - | - | - | - | - | - |
|  | *Taraxacum.officinale* | | - | - | - | 0.10 | - | 0,05 | < 0,006 | - | < 0,006 | 0,80 | - | 0,59 |
|  | *Teucrium.chamaedrys* | | 0.04 | - | 0.04 | - | 0.02 | 0,01 | - | < 0,006 | < 0,006 | - | - | - |
|  | *Thlaspi.alpestre* | | 3.45 | 2.08 | 3.31 | 0.41 | 0.15 | 0,28 | - | - | - | - | - | - |
| **Evergreen forb** | | | **3.82** | **0.58** | **3.47** | **1.02** | **0.01** | **0.49** | **0,08** | **-** | **0,04** | **0,13** | **-** | **0,09** |
|  | *Globularia.cordifolia* | | 1.22 | 0.46 | 1.14 | 0.03 | - | 0,02 | - | - | - | - | - | - |
|  | *Helianthemum.sp* | | 0.71 | 0.13 | 0.65 | 0.27 | - | 0,13 | - | - | - | - | - | - |
|  | *Polygala.chamaebuxus* | | 1.74 | - | 1.55 | 0.72 | 0.01 | 0,34 | 0,08 | - | 0,04 | 0,13 | - | 0,09 |
|  | *Thymus.serpyllum* | | 0.14 | - | 0.13 | - | - | - | - | - | - | - | - | - |
| **Deciduous shrub** | | | **0.67** | **0.81** | **0.69** | **4.12** | **1.32** | **2.66** | **1,94** | **0,96** | **1,48** | **2,41** | **1,00** | **2,03** |
|  | *Alnus.viridis* | | 0.02 | 0.67 | 0.09 | 0.07 | 0.15 | 0,11 | 0,04 | 0,11 | 0,07 | - | - | - |
|  | *Cornus.sanguinea* | | - | - | - | - | - | - | 0,09 | - | 0,05 | - | - | - |
|  | *Corylus.avellana* | | - | 0.04 | < 0.006 | - | 0.16 | 0,08 | 0,01 | 0,14 | 0,07 | 0,01 | 0,01 | 0,01 |
|  | *Cotoneaster.integerrimus* | | 0.02 | - | 0.01 | 0.01 | - | < 0,006 | - | - | - | - | - | - |
|  | *Frangula.alnus* | | - | - | - | 0.07 | - | 0,03 | 0,05 | - | 0,03 | - | - | - |
|  | *Lonicera.alpigena* | | - | - | - | 0.18 | - | 0,09 | 0,17 | - | 0,09 | 0,08 | - | 0,06 |
|  | *Lonicera.periclymenum* | | - | - | - | 1.21 | - | 0,58 | - | - | - | - | - | - |
|  | *Lonicera.xylosteum* | | 0.04 | - | 0.03 | 0.07 | - | 0,03 | 0,66 | - | 0,35 | 0,04 | - | 0,03 |
|  | *Rhamnus.alpinus* | | - | - | - | 0.06 | - | 0,03 | - | - | - | - | - | - |
|  | *Ribes.alpinum* | | 0.01 | - | 0.01 | - | - | - | 0,01 | - | < 0,006 | 0,09 | 0,23 | 0,13 |
|  | *Rosa.sp* | | 0.38 | 0.04 | 0.34 | 1.40 | 0.05 | 0,69 | 0,33 | 0,02 | 0,18 | 0,07 | - | 0,05 |
|  | *Rubus.ideaus* | | - | - | - | - | - | - | - | - | - | 0,02 | 0,04 | 0,03 |
|  | *Salix.sp* | | 0.10 | - | 0.09 | 0.07 | 0.24 | 0,16 | 0,37 | 0,03 | 0,21 | 0,03 | - | 0,02 |
|  | *Sambucus.racemosa* | | - | - | - | - | - | - | 0,06 | - | 0,03 | - | - | - |
|  | *Vaccinium.myrtillus* | | 0.11 | 0.04 | 0.10 | 0.97 | 0.72 | 0,84 | 0,16 | 0,66 | 0,40 | 2,07 | 0,73 | 1,71 |
|  | *Viburnum.lantana* | | - | 0.02 | < 0.006 | 0.02 | - | 0,01 | - | - | - | - | - | - |
| **Evergreen shrub** | | | **19.62** | **16.15** | **19.25** | **16.20** | **17.28** | **16.76** | **13,53** | **26,14** | **19,52** | **44,34** | **58,62** | **48,18** |
|  | *Arctostaphylos.uva.ursi* | | 10.28 | 4.29 | 9.63 | 5.24 | 0.49 | 2,75 | 0,56 | 0,76 | 0,66 | 0,23 | - | 0,17 |
|  | *Buxus.sempervirens* | | 0.09 | 0.40 | 0.12 | 0.01 | 0.65 | 0,34 | 0,08 | 0,04 | 0,06 | - | - | - |
|  | *Hedera.helix* | | 0.51 | 0.13 | 0.47 | 1.68 | 9.70 | 5,88 | 3,55 | 12,25 | 7,68 | 6,14 | 19,39 | 9,71 |
|  | *Juniperus.communis* | | 0.89 | - | 0.79 | 0.15 | - | 0,07 | 0,07 | 0,04 | 0,05 | - | - | - |
|  | *Ligustrum.vulgare* | | 5.78 | 5.23 | 5.72 | 5.40 | 1.26 | 3,23 | 1,33 | 1,16 | 1,25 | 2,26 | 0,08 | 1,68 |
|  | *Rhododendron.ferrugineum* | | 1.32 | 0.27 | 1.20 | 2.26 | 0.18 | 1,17 | < 0,006 | 0,12 | 0,06 | 0,02 | - | 0,02 |
|  | *Rubus.fruticosus* | | 0.76 | 5.83 | 1.31 | 1.46 | 5.01 | 3,32 | 7,94 | 11,78 | 9,76 | 35,68 | 39,14 | 36,62 |
| **Deciduous tree** | | | **0.79** | **0.48** | **0.75** | **5.81** | **2.36** | **4.01** | **4,12** | **1,54** | **2,89** | **5,70** | **0,61** | **4,33** |
|  | *Acer.campestre* | | - | - | - | - | 0.05 | 0,03 | 0,10 | - | 0,05 | 0,05 | - | 0,04 |
|  | *Acer.pseudoplatanus* | | 0.73 | 0.38 | 0.69 | 2.09 | 0.84 | 1,44 | 0,88 | 0,36 | 0,64 | 2,87 | 0,26 | 2,17 |
|  | *Betula.pendula* | | 0.03 | - | 0.03 | 0.29 | - | 0,14 | 0,01 | - | < 0,006 | - | 0,07 | 0,02 |
|  | *Castanea.sativa* | | - | - | - | - | - | - | 0,01 | - | 0,01 | - | - | - |
|  | *Crataegus.sp* | | - | - | - | - | - | - | - | 0,01 | 0,01 | - | - | - |
|  | *Fagus.sylvatica* | | < 0.006 | - | < 0.006 | 1.01 | 0.44 | 0,71 | 0,53 | 0,63 | 0,58 | 0,47 | 0,04 | 0,36 |
|  | *Fraxinus.excelsior* | | 0.02 | - | 0.02 | 0.11 | 0.93 | 0,54 | 0,74 | 0,04 | 0,41 | 0,54 | 0,21 | 0,45 |
|  | *Malus.sylvestris* | | - | 0.10 | 0.01 | 0.16 | - | 0,08 | 0,81 | 0,04 | 0,44 | 0,13 | - | 0,10 |
|  | *Populus.tremula* | | - | - | - | 0.06 | - | 0,03 | 0,01 | - | < 0,006 | 0,11 | - | 0,08 |
|  | *Prunus.avium* | | - | - | - | - | - | - | - | 0,20 | 0,09 | - | - | - |
|  | *Pyrus.pyraster* | | - | - | - | - | - | - | 0,07 | 0,03 | 0,05 | - | - | - |
|  | *Quercus.sp* | | - | - | - | 2.03 | 0.05 | 1,00 | 0,45 | 0,19 | 0,33 | 1,53 | 0,02 | 1,13 |
|  | *Sorbus.aria* | | - | - | - | 0.05 | - | 0,02 | 0,41 | 0,04 | 0,23 | - | - | - |
|  | *Sorbus.aucuparia* | | - | - | - | 0.01 | 0.05 | 0,03 | 0,10 | - | 0,05 | - | - | - |
| **Evergreen tree** | | | **0.42** | **17.50** | **2.27** | **1.45** | **28.07** | **15.38** | **4,02** | **15,69** | **9,56** | **0,80** | **10,23** | **3,34** |
|  | *Abies.alba* | | 0.26 | 15.98 | 1.96 | 1.02 | 18.41 | 10,12 | 2,89 | 9,70 | 6,12 | 0,28 | 7,12 | 2,12 |
|  | *Ilex.aquifolium* | | - | - | - | - | 0.55 | 0,29 | 0,12 | 1,17 | 0,62 | < 0,006 | 0,05 | 0,02 |
|  | *Picea.abies* | | 0.13 | 1.44 | 0.27 | 0.42 | 8.04 | 4,41 | 0,94 | 2,64 | 1,74 | 0,52 | 3,06 | 1,20 |
|  | *Pinus.nigra* | | - | - | - | - | - | - | - | 1,06 | 0,50 | - | - | - |
|  | *Pinus.sylvestris* | | - | - | - | - | - | - | 0,08 | - | 0,04 | - | - | - |
|  | *Taxus.baccata* | | 0.03 | 0.08 | 0.04 | - | 1.07 | 0,56 | < 0,006 | 1,12 | 0,54 | - | - | - |
| **Bark** |  | | **-** | **-** | **-** | **0.14** | **0.33** | **0,24** | **0,10** | **0,22** | **0,16** | **-** | **-** | **-** |
| **Bryophyta** | | | **0.01** | **-** | **0.01** | **-** | **0.02** | **0.01** | **0,01** | **0,01** | **0,01** | **0,05** | **0,06** | **0,05** |
| **Dead leaf** | | | **1.35** | **0.90** | **1.30** | **2.63** | **3.13** | **2.90** | **3,31** | **2,40** | **2,87** | **2,83** | **3,38** | **2,98** |
| **Epiphyte** | | *Viscum.album* | **-** | **-** | **-** | **0.01** | **0.24** | **0,13** | **0,61** | **0,63** | **0,62** | **0,04** | **0,13** | **0,06** |
| **Lichen** | |  | **< 0.006** | **-** | **< 0.006** | **0.01** | **0.17** | **0,09** | **-** | **-** | **-** | **-** | **0,02** | **0,01** |
| **Mushroom** | | | **0.02** | **-** | **0.02** | **0.53** | **0.03** | **0.27** | **0,22** | **0,04** | **0,13** | **2,93** | **0,30** | **2,22** |
| **Woody debris** | | | **1.69** | **1.54** | **1.67** | **4.87** | **7.41** | **6.20** | **6,13** | **4,63** | **5,42** | **6,52** | **3,35** | **5,66** |
| **Unknown** | | | **0.84** | **1.10** | **0.86** | **0.52** | **0.09** | **0.29** | **0,12** | **0,04** | **0,08** | **0,08** | **0,32** | **0,15** |

**Figure S3.** Relationship between diet’s composition and quality for the two periods performed using co-inertia analysis on two PCA (abundance per plant type in rumen and chemical content as hemicellulose (Hem), lignin (Lig), cellulose (Cel), soluble fraction (SF) and nitrogen (N) in rumen). The 3 chemical components and the plant types contributing the most to the axes are displayed as arrows allowing axes interpretation. Absolute lengths of the arrows are arbitrary and chosen to display well on the figure.

Roe deer


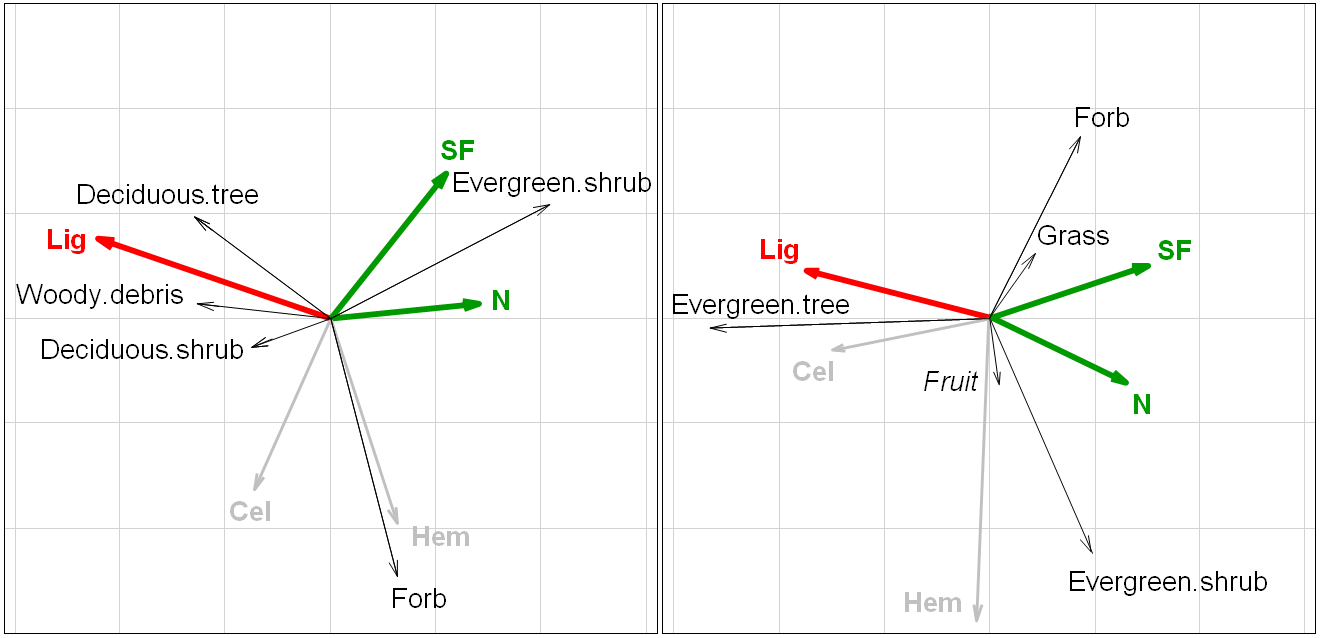


Chamois


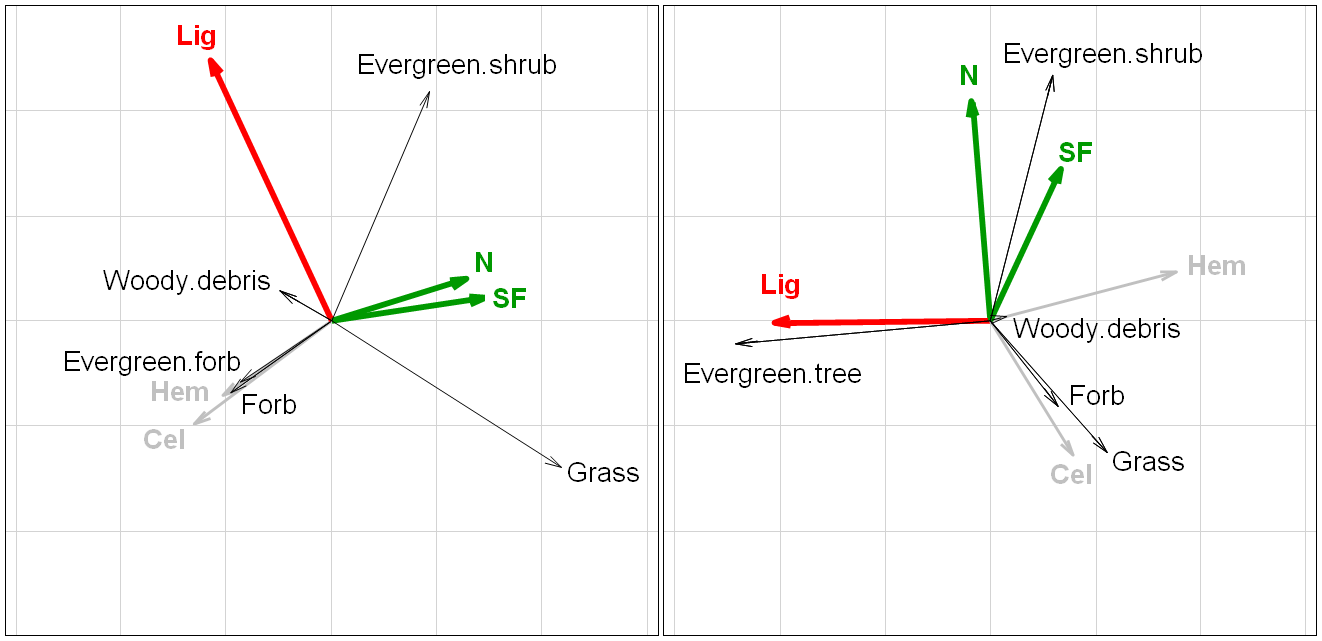


Mouflon


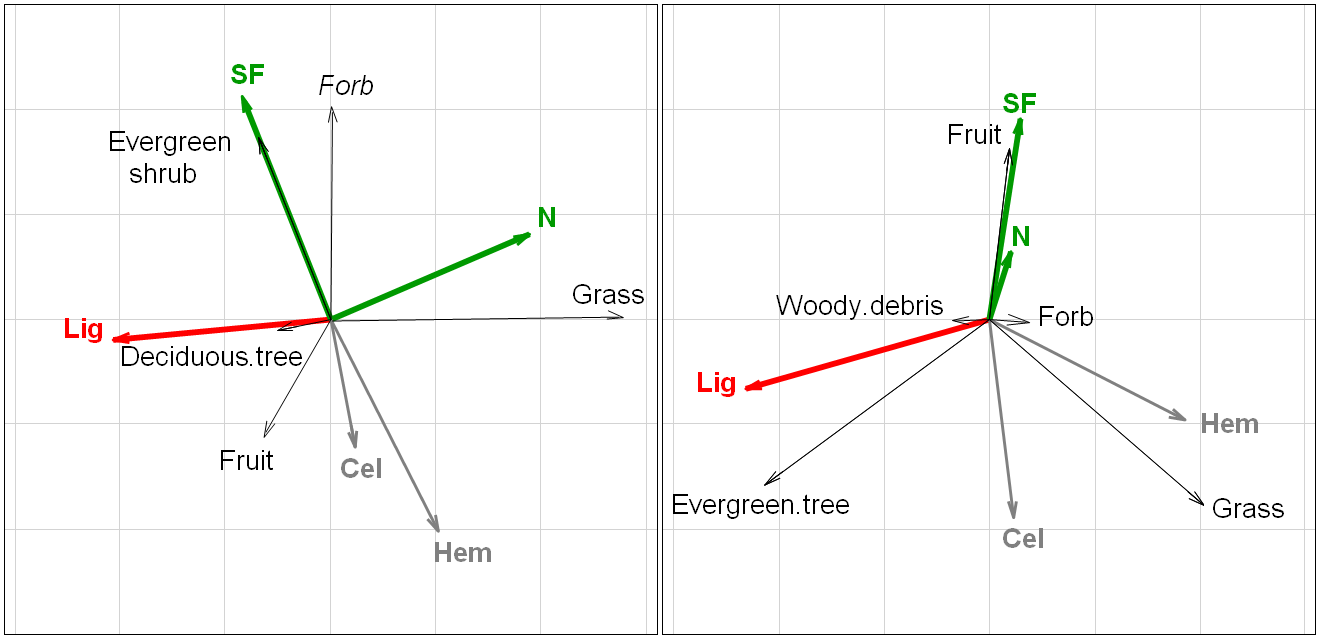


Red deer


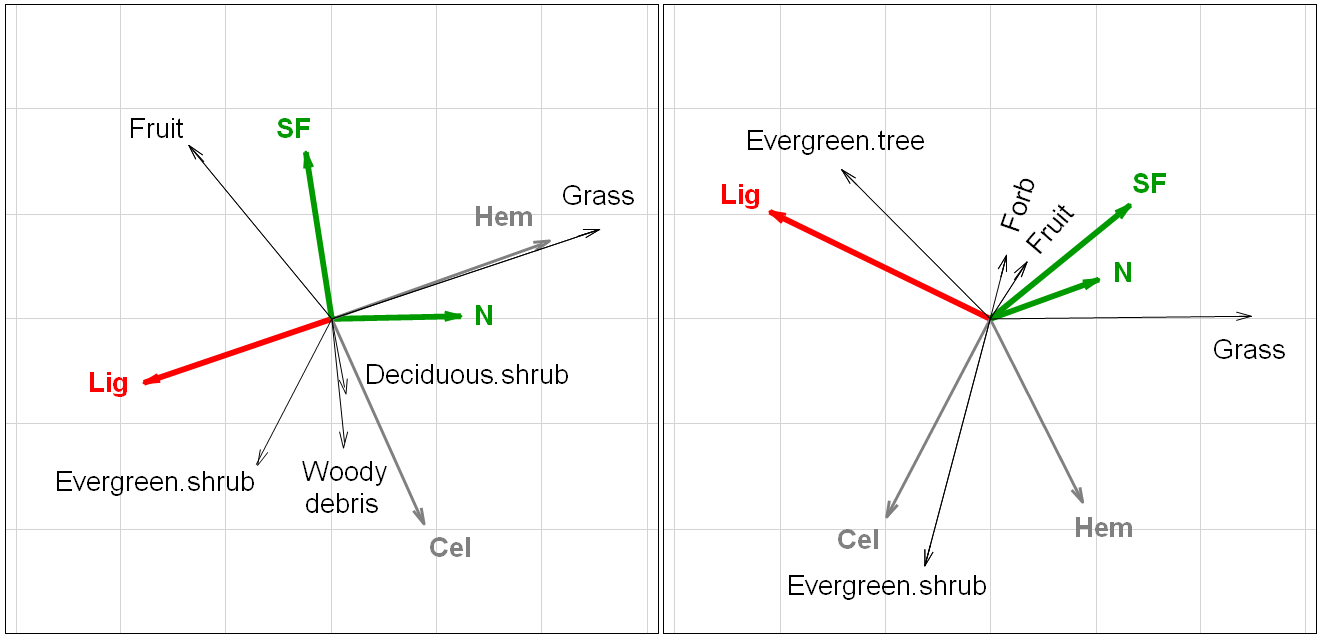


**Table S3.** Best models for the analyses of the relationship between grass content in the diet and lignin, hemicellulose, nitrogen, cellulose and soluble fraction (sol. fraction) contents. Models with AIC within 2 units of the model with the lowest AIC are presented with their number of parameters, ΔAIC (difference with the best model), and AIC weight . Among the models with close AIC values, we selected the model with the lowest number of parameters. Figures with the predicted values is in the main body of the text (Fig. 5)

|  | Grass*Species | Grass*Period | Species*Period | Grass | Species | Period | df | ΔAIC | AIC weight |
| --- | --- | --- | --- | --- | --- | --- | --- | --- | --- |
| Lignin | **X** |  |  | **X** | **X** | **X** | **10** | **0** | **0.406** |
|  | x | x |  | x | x | x | 11 | 1.91 | 0.157 |
| Hemicellulose | **X** |  | **X** | **X** | **X** | **X** | **13** | **0** | **0.466** |
|  | x | x | x | x | x | x | 14 | 1.91 | 0.179 |
| Nitrogen |  |  | x | x | x | x | 10 | 0 | 0.349 |
|  |  |  |  | **X** | **X** | **X** | **7** | **0.54** | **0.267** |
|  |  | x |  | x | x | x | 11 | 1.54 | 0.161 |
| Cellulose |  |  |  |  | **X** | **X** | **6** | **0** | **0.280** |
|  |  |  |  | x | x | x | 7 | 0.77 | 0.191 |
|  |  |  | x |  | x | x | 9 | 1.76 | 0.116 |
| Sol. fraction | x |  | x | x | x | x | 13 | 0 | 0.27 |
|  |  |  | **X** | **X** | **X** | **X** | **10** | **0.39** | **0.22** |
|  |  | x | x | x | x | x | 14 | 0.79 | 0.181 |
